# Environmental and sensitization variations among asthma and/or rhinitis patients between 2008 and 2018 in China

**DOI:** 10.1002/clt2.12116

**Published:** 2022-02-02

**Authors:** Wanjun Wang, Jianhong Wang, Guihua Song, Hua Xie, Xiaoping Lin, Ruonan Chai, Rongfei Zhu, Yong He, Jun Tang, Junge Wang, Jinghua Yang, Lili Zhi, Lin Wu, Yan Jiang, Xiaoqin Zhou, Dongming Huang, Ning Wang, Rui Xu, Yuan Gao, Zhimin Chen, Jinling Liu, Xiaoli Han, Guolin Tan, Jinzhun Wu, Deyu Zhao, Jianjun Chen, Xiwei Zhang, Mengrong Li, Yuemei Sun, Yi Jiang, Weitian Zhang, Qianhui Qiu, Chuanhe Liu, Jie Yin, Guodong Hao, Huabin Li, Yongsheng Xu, Shaohua Chen, Hua Zhang, Shi Chen, Juan Meng, Dan Zeng, Wei Tang, Chuangli Hao, Jing Li, Nanshan Zhong, for the China Alliance of Research on Respiratory Allergic Disease

**Affiliations:** ^1^ Department of Allergy and Clinical Immunology National Clinical Research Center for Respiratory Disease State Key Laboratory of Respiratory Disease Guangzhou Institute of Respiratory Health, The First Affiliated Hospital of Guangzhou Medical University Guangzhou China; ^2^ The First People's Hospital of Yibin Yibin Sichuan China; ^3^ The First Affiliated Hospital of Henan University of Traditional Chinese Medicine Zhengzhou Henan China; ^4^ General Hospital of Northern Theater Command Shenyang China; ^5^ Tongji Hospital Tongji Medical College Huazhong University of Science & Technology Wuhan China; ^6^ The Affiliated Hospital of Medical School Ningbo University Ningbo Zhejiang China; ^7^ Foshan First People's Hospital Foshan China; ^8^ Beijing Hospital of Traditional Chinese Medicine Beijing China; ^9^ Guangdong Provincial Hospital of Chinese Medicine Guangzhou China; ^10^ The First Affiliated Hospital of Shandong First Medical University Shandong Institute of Respiratory Diseases Taian China; ^11^ Hangzhou Hospital of Traditional Chinese Hangzhou China; ^12^ The Affiliated Hospital of Qingdao University Qingdao China; ^13^ Hubei Province Maternal and Child Health Hospital Wuhan Hubei China; ^14^ Boai Hospital of Zhongshan City Zhongshan China; ^15^ Xi'an Children's Hospital Shaanxi China; ^16^ The First Affiliated Hospital of Sun‐Yat University Guangzhou China; ^17^ The First Affiliated Hospital of Zhengzhou University Zhengzhou China; ^18^ Children's Hospital of Zhejiang University School of Medicine National Clinical Research Center for Child Health Hangzhou China; ^19^ Hebei General Hospital Hebei China; ^20^ Third Xiangya Hospital of Central South University Changsha China; ^21^ The Women and Children's Hospital Affiliated to Xiamen University Xiamen China; ^22^ Children's Hospital of Nanjing Medical University Nanjing China; ^23^ Union Hospital of Tongji Medical College Wuhan China; ^24^ The Second Affiliated Hospital and Yuying Children's Hospital of Wenzhou Medical University Wenzhou China; ^25^ Yu Huang Ding Hospital Yantai China; ^26^ The First Hospital of Shanxi Medical University Taiyuan China; ^27^ Shanghai Jiao Tong University Affiliated Sixth People's Hospital Shanghai China; ^28^ Zhujiang Hospital of Southern Medical University Guangzhou China; ^29^ Children's Hospital Capital Institute of Pediatrics Beijing China; ^30^ Chengdu First People's Hospital Foshan China; ^31^ Tangshan Gongren Hospital Hebei China; ^32^ ENT Institute and Department of Otorhinolaryngology Eye & ENT Hospital Fudan University Shanghai China; ^33^ Children's Hospital of Tianjin University Tianjin China; ^34^ Guangdong Provincial People's Hospital Guangzhou China; ^35^ The First Affiliated Hospital of Xinjiang Medical University Ürümqi China; ^36^ Hainan Provincial People's Hospital Haikou China; ^37^ West China Hospital of Sichuan University Chengdu China; ^38^ Chongqing General Hospital University of Chinese Academy of Sciences Beijing China; ^39^ Ruijin Hospital of Shanghai Jiaotong University Shanghai China; ^40^ Children's Hospital of Soochow University Suzhou China

**Keywords:** aeroallergens, aeroallergens, anaphylaktische reaktionen, asthma, asthma, environment, epidemiologie, epidemiology, rhinitis, rhinitis, sensitization, Stichworte, umwelt

## Abstract

**Background:**

Little is known about the changes in allergen sensitization in China secondary to the environmental variations over the past decade. We aimed at investigating the variations in sensitization among asthma and/or rhinitis patients in China between 2008 and 2018.

**Methods:**

This study analyzed cross‐sectional data from national surveys conducted in China in 2008 and 2018. After finishing the questionnaire, participants underwent serum specific IgE measurements. A total of 2322 and 2798 patients were enrolled in 2008 and 2018, respectively. The significance of differences in sensitization rates among four regions of China were assessed. Correlation analysis was used to identify the associations of sensitization with climate change and planting of *Artemisia desertorum* between the two surveys.

**Results:**

Compared with 2008, the general sensitization rate to mites significantly increased in 2018, which ranked highest among all tested allergens. Sensitization to pollens, especially *Artemisia vulgaris*, showed the greatest increase in the north. The annual mean temperature, rainfall and relative humidity in all four regions, and the *Artemisia desertorum* coverage in the northeastern area, increased significantly in 2018 as compared with 2008. From 2008 to 2018, an increase in *Dermatophagoides pteronyssinus* sensitization was significantly associated with an increase in relative humidity (*r* = 0.54, *p* = 0.037). The increase in *A. vulgaris* sensitization was significantly associated with the increase in the *A. desertorum* planting area (*r* = 0.67, *p* = 0.006) and with a decrease in rainfall (*r* = −0.59, *p* = 0.021).

**Conclusions:**

House dust mites remain the most important allergen in Chinese individuals with asthma and/or rhinitis. Pollen sensitization dramatically increased in northern China. Increases in sensitization to dust mites and Artemisia were related to the increases in humidity and planting area of *A. desertorum*.

## 1 INTRODUCTION

Recent epidemiological studies revealed that the prevalence of allergic rhinitis and asthma dramatically increased in China.^1^ ^,^ ^2^ Exposure to aeroallergens is one of the crucial factors in inducing sensitization and affects development of asthma and rhinitis.^3^, ^4^ In 2008, to better represent sensitization rates in rhinitis and asthma, the China Alliance of Research on Respiratory Allergic Disease (CARRAD) conducted a national cross‐sectional study on allergen sensitization in outpatients with asthma and rhinitis. The study demonstrated that house dust mites were the most prevalent allergens in China. The allergen sensitization rates varied by geographic location.^5^ Although the results of this survey provided a reference for allergy and clinical immunology in China, the environmental factors underlying the variations in the prevalence of allergen sensitization remain unknown. In follow‐up, regional epidemiological studies on sensitization rates of airway diseases were performed.^6^ ^,^ ^7^ ^,^ ^8^ However, those studies had limited value being locoregional in scope and not defining precisely disease states among study subjects.

One decade has passed since our last national multi‐center epidemiologic survey in 2008. Since then, tremendous transformations have taken place in China such as more westernized living and working styles, urbanization, and increases in temperature and humidity. Especially in the north, regional governments have made great efforts to plant a large scale of *Artemisia desertorum* in order to control the impact of sandstorms on large cities. Little is known about the impact of social and environmental changes on temperature and humidity as well as sensitizations in the general population or in patients with rhinitis and/or asthma. Given the needs in providing important evidence for national developmental strategies and clinical managements to reduce the burden and morbidity of allergic airway diseases, CARRAD decided to conduct a second national survey in 2018 with the same study protocol used in 2008 to understand environmental and sensitization variations among patients with rhinitis and asthma in the past 10 years. Herein, we tested the hypothesis that sensitization rates have increased among individuals with asthma and/or rhinitis in China between the years 2008 and 2018.

## 2 METHODS

### 2.1 Study participants and survey design

The 2008 survey was conducted from January 2008 to December 2008 in 17 cities with 24 participating centers. Expanding on this, the 2018 study was conducted from January 2018 to December 2018 in 26 cities with 36 centers from the northern, eastern, central, and southern coastal regions of China. A total of 6345 patients and 5532 patients, aged 5–65 years, in outpatient clinics at the study sites were enrolled in 2008 and 2018, respectively. The studies covered mid‐temperate, warm‐temperate, subtropical, and tropical zones of China. Uniform protocol, questionnaire, allergen testing set, and operating procedures were used among all study centers. Questionnaire interviewers were trained before the study. Results of the questionnaire and blood tests were sent monthly to one study center for data analysis. Quality control reports were then prepared for all study centers. At multiple levels data were verified by the investigators. All questionnaire and blood test data were coded and input into a programmed database by two workers. The entered data were checked for out‐of‐range values and logic mistakes. The study protocol was approved by the ethics review board of each study center. All the participants gave written informed consent to participate in the study. Details about the survey participants are found in Appendix S1.

## 3 OUTCOME MEASURES

Patients with rhinitis, asthma, and asthma with rhinitis were identified by evaluation of history, questionnaire, and relevant tests. Rhinitis was defined as symptoms of sneezing, or a running, itchy, or blocked nose, in the absence of a cold or flu.^9^ Asthma was defined as a history of recurrent dyspnea, wheezing, or cough episodes; positive airway reversibility testing (FEV_1_ increasing ≥12% and 200 ml after inhalation of 400 mg of salbutamol); or positive airway responsiveness testing (FEV_1_ decreasing ≥20% upon administration of ≤7.8 μg of histamine).^10^ The 2008 and 2018 studies employed the same standardized questionnaire to record demographic characteristics; family history of allergic diseases; symptoms of the airways, skin, and eyes; smoking habits; socio‐economic conditions (e.g., gender, age, number and age of siblings, education, and social status); and environment exposures and dietary habits.^11^ The patients were interviewed face to face by the research physicians or nurses. Questions about the impact of allergic symptoms on daily activities, working and schooling, night‐time sleep, and use of medications for controlling the symptoms were also included.

Additionally, a peripheral blood sample of 10 ml was taken from each subject, coagulated at room temperature, centrifuged, stored at −20 °C, and sent to a central laboratory in the first affiliated hospital of Guangzhou Medical University. Specific IgE antibodies (sIgE) against *Dermatophagoides pteronyssinus*, *Dermatophagoides farina*, *Blomia tropicalis*, cat dander, dog dander, timothy grass, *Populus nigra*, *Ambrosia artemisifolia*, *Artemisia vulgaris*, and *Alternaria alternata* were measured on an ADVIA Centaur immunoassay system (Siemens AG, Erlangen, Germany). The analysis of sIgE was performed only in patients in whom allergen testing was done. The results were categorized into the following groups: grade 0 (<0.35 IU/ml), grade 1 (0.35–0.70 IU/ml), grade 2 (0.70–3.50 IU/ml), grade 3 (3.5–17.5 IU/ml), grade 4 (17.5–50 IU/ml), grade 5 (50–100 IU/ml), and grade 6 (>100 IU/ml). The cutoff value was set at 0.35 IU/ml. The response was defined as positive if the sIgE level was ≥0.35 IU/L.

## 4 STATISTICAL ANALYSIS

According to the region and sex distributions in age groups in 2008, the population in the 2018 survey was randomly sampled and adjusted for 8 age groups (5–7, 8–10, 11–14, 15–24, 25–34, 35–44, 45–54, and 55–65 years). Between‐group differences in baseline characteristics were compared using standardized differences before and after stratified sampling.^12^ Changes in temperature, humidity, rainfall, and *A. desertorum* coverage were treated as continuous variables. Geographical area, children age groups, adult age groups, and disease groups were treated as categorical variables. We assessed the significance of differences using ANOVA for continuous variables and the *χ* ^2^ test for categorical variables. The differences in sensitization rates in children and adults between 2008 and 2018 were determined by the Pearson *χ* ^2^ test. Fisher's exact test was used to determine the association between the categorical variables. The annual mean temperature, humidity, and rainfall of the different geographical regions were provided by the Earth Science Data Sharing Platform (http://www.geodata.cn) and the China Meteorological Data Service Center (http://data.cma.cn). The data on the distribution of *A. desertorum* were provided and estimated by the Chinese Virtual Herbarium (http://www.cvh.org.cn), National Forestry and Grassland Data Center (https://www.forestdata.cn), Flora of China (https://www.iplant.cn), and Annals of China Forestry and Grassland statistics. The site coordinates were imported into the ArcGIS10.5 software, and the relevant nonspatial information was constructed. In this step, we downloaded the vector boundary of China administrative region (1:4,000,000) to limit the scope of interpolation analysis, and finally the interpolation results were visualized and mapped. The associations of climate change and *A. desertorum* planting with *D. pteronyssinus* and *A. vulgaris* sensitization were assessed using Spearman rank correlation. All the data were analyzed using the Statistical Package for the Social Sciences for Windows, release 21.0 (SPSS, Inc., Chicago, IL). *p* Value less than 0.05 was considered statistically significant.

## 5 RESULTS

### 5.1 Demographic data

In 2008, 2322 participants, including 810 individuals with rhinitis, 617 with asthma, and 895 asthmatics with rhinitis, completed the questionnaire and underwent serum IgE test. In 2018, 2798 participants were randomly selected in the final analysis, including 1871 with rhinitis, 140 with asthma, and 787 asthmatics with rhinitis. After stratified sampling, the two groups were balanced in terms of gender, age group, and geographical region (Table 1).

**TABLE 1:** Demographic characteristics of the study population

	2008	2018^a^	Standardized difference (%)^d^	2008	2018^b^	Standardized difference (%)^d^
No	2322	4317		2322	2798	
Gender, No (%)						
Male	1195 (51.5)	2136 (49.5)	4	1195 (51.5)	1455 (52.0)	1
Female	1127 (48.5)	2181 (50.5)	4	1127 (48.5)	1343 (48.0)	1
Children^c^	1007 (43.4)	1826 (42.3)	2	1007 (43.4)	1212 (43.3)	0
Adults	1315 (56.6)	2491 (57.7)	2	1315 (56.6)	1586 (56.7)	0
Age group, No.(%), Yr						
5–7	494 (21.3)	900 (20.8)	1	494 (21.3)	587 (21.0)	0
8–10	323 (13.9)	578 (13.4)	1	323 (13.9)	412 (14.7)	2
11–14	190 (8.2)	348 (8.1)	0	190 (8.2)	213 (7.6)	2
15–24	233 (10.0)	505 (11.7)	5	233 (10.0)	291 (10.4)	1
25–34	401 (17.3)	911 (21.1)	11	401 (17.3)	518 (18.5)	6
35–44	336 (14.5)	620 (14.4)	0	336 (14.5)	402 (14.4)	0
45–54	226 (9.7)	300 (6.9)	10	226 (9.7)	251 (9.0)	2
55–65	119 (5.1)	155 (3.6)	7	119 (5.1)	124 (4.4)	3
Region, No. (%)						
North						
Children	252 (10.9)	545 (12.6)	5	252 (10.9)	300 (10.7)	0
Adults	607 (26. 1)	909 (21.1)	12	607 (26. 1)	704 (25.2)	2
Total	859 (37.0)	1454 (33.7)	7	859 (37.0)	1004 (35.9)	2
East						
Children	151 (6.5)	491 (11.4)	17	151 (6.5)	222 (7.9)	5
Adults	109 (4.7)	255 (5.9)	5	109 (4.7)	145 (5.2)	2
Total	260 (11.2)	746 (17.3)	18	260 (11.2)	367 (13.1)	6
Central						
Children	187 (8.1)	296 (6.9)	5	187 (8.1)	260 (9.3)	4
Adults	196 (8.4)	665 (15.4)	22	196 (8.4)	251 (9.0)	2
Total	383 (16.5)	961 (22.3)	15	383 (16.5)	511 (18.3)	5
South						
Children	417 (18.0)	494 (11.4)	19	417 (18.0)	430 (15.4)	7
Adults	403 (17.4)	662 (15.3)	6	403 (17.4)	486 (17.4)	0
Total	820 (35.3)	1156 (26.8)	18	820 (35.3)	916 (32.7)	5

^a^ Before stratified sampling.

^b^ After stratified sampling.

^c^ Referred to as 5–14 years old.

^d^ The difference between the groups divided by the pooled standard deviation; a value greater than 10% is interpreted as a meaningful difference.^12^

### 5.2 Comparison of allergen sensitization rates

As shown in Table 2, the general sensitization rates to mites were significantly higher in 2018 than in 2008 (*D. pteronyssinus, D. farinae, B. tropicalis*: 52.1% vs. 45.4%, 51.7% vs. 44.5%, 30.7% vs. 24.1%, respectively; all *p* < 0.01). Likewise, sensitization rates to pollens (*P. nigra*, *A. artemisifolia*, and *A. vulgaris*; 4.2% vs. 2.9%, 5.2% vs. 1.7%, 10.5% vs. 7.4%, respectively; all *p* < 0.05) and dog fur (5.9% vs. 3.7%, *p* < 0.01) was increased in 2018 compared with 2008. No significant differences in sensitization rates to cat dander, timothy grass, and *A. alternata* were observed between the 2018 and 2008 cohorts.

**TABLE 2:** Comparison of allergen sensitizations between 2008 and 2018 in children and adults in four regions of China

Regions of China	*n* (%)	Year of study	*Dermatophagoides pteronyssinus*	*Dermatophagoides farinae*	*Blomia tropicalis*	Cat dander	Dog dander	Timothy grass	*Populus nigra*	*Ambrosia artemisifolia*	*Artemisia vulgaris*	*Alternaria alternata*
	Total	2008	1054 (45.4)	1033 (44.5)	559 (24.1)	197 (8.5)	85 (3.7)	41 (1.8)	68 (2.9)	40 (1.7)	169 (7.4)	163 (7.0)
		2018	1458 (52.1)‡	1446 (51.7)‡	860 (30.7)‡	203 (7.3)	166 (5.9)‡	74 (2.6)	117 (4.2)†	146 (5.2)‡	295 (10.5)‡	169 (6.0)
		2008	633 (62.7)**	618 (61.4)**	334 (33.2)**	105 (10.4)**	17 (1.7)**	20 (2.0)	30 (3.0)	10 (1.0)*	30 (3.0)**	130 (12.9)**
General	Children	2018	793 (65.4)**	789 (65.1)**	421 (34.7)**	106 (8.7)**	76 (6.3)‡	33 (2.7)	68 (5.6)‡**	56 (4.6)‡	82 (6.8)‡**	145 (12.0)**
		2008	421 (32.0)	415 (31.6)	225 (17.1)	92 (7.0)	68 (5.2)	21 (1.6)	38 (2.9)	30 (2.3)	139 (10.6)	33 (2.5)
	Adults	2018	665 (41.9)‡	657 (41.4)‡	439 (27.7)‡	97 (6.1)	90 (5.7)	41 (2.6)	49 (3.1)	90 (5.7)†	213 (13.4)†	24 (1.5)
		2008	260 (30.3)	248 (28.9)	135 (15.7)	56 (6.5)	30 (3.5)	20 (2.4)	22 (2.6)	19 (2.2)	87 (10.1)	48 (5.6)
	Total	2018	392 (39.0)^‡^	375 (37.4)^‡^	207 (20.6)^‡^	82 (8.1)	70 (7.0)^‡^	68 (6.8)^‡^	94 (9.3)^‡^	116 (11.6)^‡^	272 (27.1)^‡^	61 (6. 1)
		2008	138 (54.8)**	135 (53.6)**	59 (23.4)**	26 (10.3)**	4 (1.6)	5 (2.0)	5 (2.0)	2 (0.8)	23 (9.1)	27 (10.7)**
North	Children	2018	169 (56.4)**	167 (55.8)**	82 (27.3)**	35 (11.5)*	23 (7.6)^‡^	25 (8.2)^‡^	38 (12.7)^‡^*	37 (12.4)^‡^	59 (19.7)^‡^**	47 (15.5)**
		2008	122 (20.1)	113 (18.6)	76 (12.5)	30 (4.9)	26 (4.3)	15 (2.5)	17 (2.8)	17 (2.8)	64 (10.5)	21 (3.5)
	Adults	2018	222 (31.6)^‡^	208 (29.5)^‡^	125 (17.7)	47 (6.7)	47 (6.7)	44 (6.2)†	56 (7.9)^‡^	79 (11.2)^‡^	213 (30.3)^‡^	15 (2. 1)
		2008	127 (48.9)^α^′	121 (46.5)^α^′	71 (27.1)^α^′	29 (11. 1)^α^	20 (7.6)^α^′	8 (2.9)	14 (5.2)^α^	0 (0.0)^α^′	11 (4.2)^α^′	30 (11.5)^α^′
	Total	2018	203 (55.2)α′	195 (53.1)α′	115 (31.5)α′	19 (5.1)^‡^	22 (5.9)	8 (2.3)α′	16 (4.3)α′	5 (1.4)α′	12 (3.1)α′	36 (9.8)α
		2008	95 (62.9)**	92 (60.9)**	50 (32.8)*^α^	11 (7.3)**	4 (2.7)**	0 (0.0)**	2 (1.2)**	0 (0.0)	2 (1.3)**^α^′	27 (18.2)**^α^
East	Children	2018	147 (66.3)**α	145 (65.2)**α	73 (33.0)	8 (3.6)α′	13 (5.8)	4 (1.7)α′	4 (1.7)**α ′	2 (1.0)α′	3 (1.4)**α ′	33 (14.9)**
		2008	32 (29.4)α	29 (26.6)α	21 (19.3)	18 (16.3)α′	16 (14.3)α′	8 (7.0)α′	12 (10.8)α′	0 (0.0)α′	9 (8.3)	3 (2.3)
	Adults	2018	55 (38.2)	50 (34.5)	42 (29.1)^α^′	11 (7.3)^‡^	9 (6.1)^‡^	4 (3.1)	12 (8.4)	3 (1.9)^α^′	8 (5.8)^α^′	3 (1.9)
		2008	182 (47.6)^α^′	176 (46.0)^α^′	105 (27.4)^α^′	32 (8.3)	16 (4.2)	6 (1.5)	11 (2.8)	3 (0.8)^α^′	25 (6.4)^α^	6 (1.5)^α^′
	Total	2018	296 (57.9)^‡α^′	285 (55.7)^‡α^′	185 (36.2)^‡^α′	28 (5.5)	24 (4.7)	6 (1.2)α′	7 (1.4)α′	9 (1.8)α′	17 (3.4)α′	22 (4.3)†
		2008	(61.5)**	112 (59.9)**	62 (33.2)**^α^	15 (8.1)	3 (1.7)*	3 (1.5)	7 (3.6)	0 (0.0)	7 (3.7)*α	6 (3.0)*^α’β’^
Central	Children	2018	168 (64.8)**^α^	165 (63.5)**^α^	98 (37.8)^α^′	23 (8.7)**	16 (6. 1)^†^	2 (0.9)^α^′	5 (1.8)^α^′	2 (0.9)^α^′	4 (1.4)*^α^′	22 (8.5)^‡^**^α’β’^
		2008	67 (34.4)^α^′	64 (32.8)α′	43 (21.8)α′	17 (8.5)β′	13 (6.5)β	3 (1.5)β′	4 (2.0)β′	3 (1.5)	18 (9.0)	0 (0.0)α′
	Adults	2018	128 (50.8)^‡α’β’^	119 (47.6)^‡^α’β’	87 (34.6)^‡^α′	6 (2.2)^‡^ αβ	8 (3.2)	4 (1.5)α′	3 (1.0)α’β’	7 (2.8)α′	14 (5.5)α′	0 (0.0)α
		2008	500 (61.0)^α’β’γ^′	487 (59.4)^α^′^β^′^γ^′	248 (30.3)α′	90 (11.0)α	25 (3.1)β′	12 (1.5)	26 (3.2)	19 (2.4)	47 (5.7)α′	52 (6.3)
Southern coast	Total	2018	642 (70.1)^‡α^′^β^′^γ^′	629 (68.7)^‡α^′^β^′^γ^′	416 (45.5)^‡α^′^β^′^γ^′	67 (7.3)^‡^	46 (5. 1)†	6 (0.7)^α^′	16 (1.7)^α^′	23 (2.5)^α^′	8 (0.9)^‡α^′^β^′^γ^′	27 (2.9)^‡α^′
		2008	299 (71.7)**^α’βγ^	293 (70.3)**^α’βγ^	162 (38.9)**^α’βγ^	60 (14.4)**^βγ^	7 (1.7)*	11 (2.7)**α4 (0.8)^α^′	17 (4.1)	10 (2.4)	20 (4.7)^α^	50 (11.9)**^γ^′
	Children	2018	341 (79.2)^‡^**^α^′^β^′^γ^′	335 (78.0)^‡^**^α β’γ^	210 (48.8)^‡**α ’β’γ^′	37 (8.6)^‡^	21 (4.9)^‡^	3 (0.6)^‡α^′	8 (1.8)^‡α^′	8 (1.8)^α^′	3 (0.8)^‡α^′	3 (0.8)^‡α^′
		2008	201 (49.9)^α^′^β^′^γ^′	194 (48.2)^α^′^β^′^γ^′	86 (21.4)α′	30 (7.5)β′	18 (4.5)β′	1 (0.3)α’β’	9 (2.3)β′	9 (2.3)β′	27 (6.8)α	2 (0.6)α′
	Adults	2018	302 (62.1)^‡α^′^β^′^γ^′	294 (60.5)^‡α^′^β^′^γ^′	207 (42.5)^‡α^′^β^′^γ^	30 (6.2)γ	25 (5.2)	4 (0.8)^α^′	8 (1.7)^α^′^β^′	16 (3.2)^α^	4 (0.9)^‡α^′^β^′^γ^′	8 (1.7)

†*p* < 0.05, ^‡^ *p* < 0.01 compared with 2008; *p < 0.05, **p < 0.01 compared with adults; α*p* < 0.05, α′*p* < 0.01 compared with North; β*p* < 0 .05, β′*p* < 0 .01 compared with East; γ*p* < 0 .05, γ′*p* < 0 .01 compared with Central.

Adults showed a greater increase in sensitization rate to mites (all *p* < 0.01), while children had a greater increase in sensitization rates to dog dander, *P. nigra*, *A. artemisifolia*, and *A. vulgaris* (all *p* < 0.01). Sensitivity to *A. alternata* was significantly higher in children than in adults in both cohorts (both *p* < 0.01).

Among four regions, individuals from the southern coast showed the highest and the north the lowest prevalence of sensitization to *D. pteronyssinus* in 2008 and 2018. Except for the east region, the other three regions showed a significant increase in sensitization rates to *D. pteronyssinus* and *D. farinae* in 2018. Sensitization to cat and *A. alternata* was higher in children than in adults in both cohorts. The northern region showed a dramatic increase in sensitization rates to *A. artemisifolia* and *A. vulgaris* in 2018 compared with those in 2008, and it showed the highest sensitization rate among the four regions in both surveys.

### 5.3 Comparison of allergen sensitization grades

**FIGURE 1:**
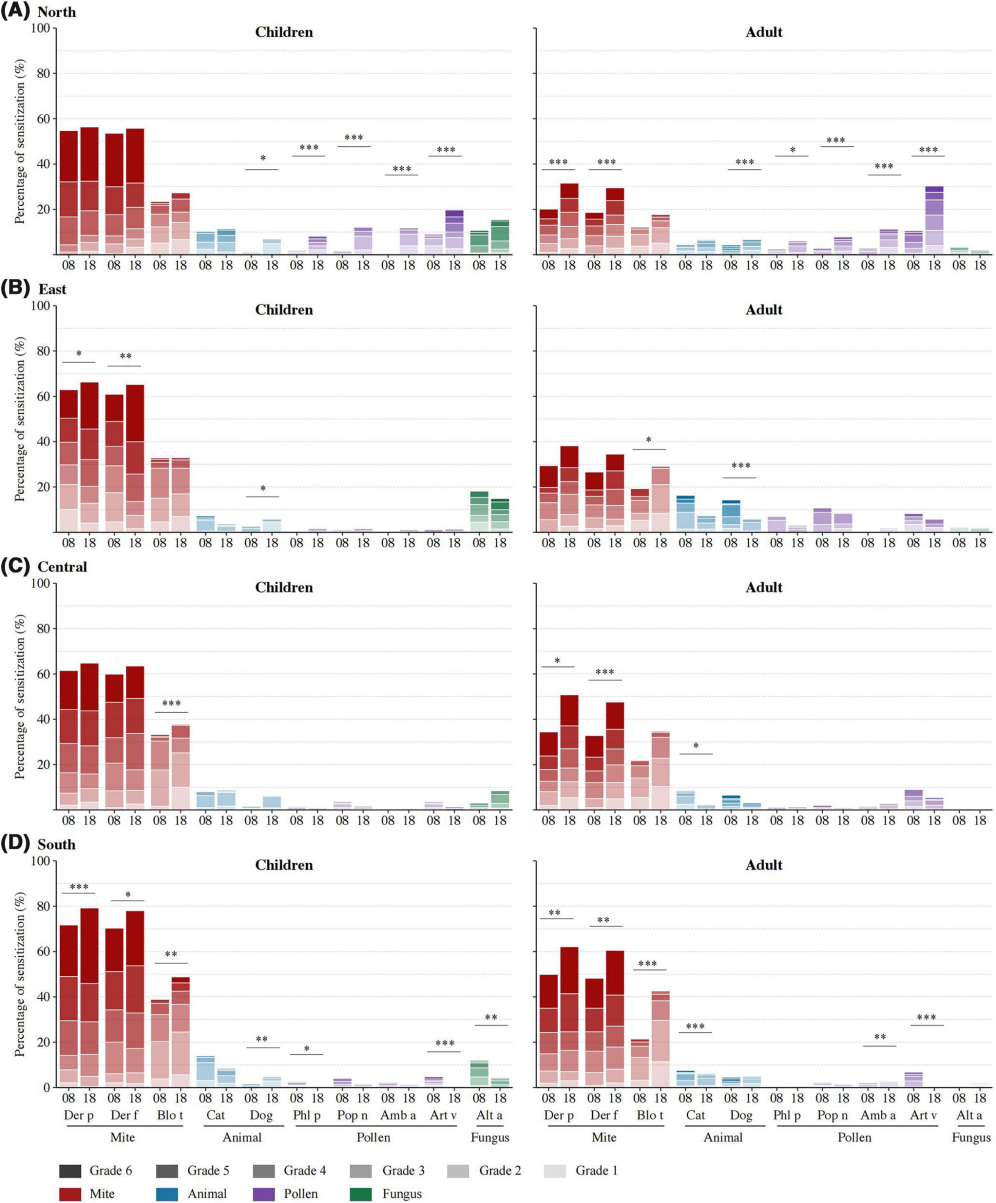
Comparison of allergen sensitization grades in children and adults in four geographic regions of China. Northern children: 2008 (*n* = 252), 2018 (*n* = 300); Northern adults: 2008 (*n* = 607), 2018 (*n* = 704); Eastern children: 2008 (*n* = 151), 2018 (*n* = 222); Eastern adults: 2008 (*n* = 109), 2018 (*n* = 145); Central children: 2008 (*n* = 187), 2018 (*n* = 260); Central adults: 2008 (*n* = 196), 2018 (*n* = 251); Southern children: 2008 (*n* = 417), 2018 (*n* = 430); Southern adults: 2008 (*n* = 403), 2018 (*n* = 486); Allergen abbreviations: Der p, *Dermatophagoides pteronyssinus*; Der f, *Dermatophagoides farinae*; Blo t, *Blomia tropicalis*; Phl p, *Phleum pretense*; Pop n, *Populus nigra*; Amb a, *Ambrosia artemisifolia*; Art v, *Artemisia vulgaris*; Alt a, *Alternaria alternata*; *p < 0·05, **p < 0·01, ***p < 0·001

Compared to 2008, results from 2018 found that in northern China, high‐degree pollen sensitization (IgE grade 4–6) and low‐degree sensitization to dog dander increased in children and adults, whereas high‐degree mite sensitization increased only in adults (all *p* < 0.05; Figure 1A). In eastern China, grade 6 sIgE levels to mites (*D. pteronyssinus* and *D. farinae*) were observed in greater numbers of children (both *p* < 0.05). High‐level sIgE sensitization to dog dander declined in adults (*p* < 0.001; Figure 1B). In central China, mite (*D. pteronyssinus* and *D. farinae*) sIgE levels (grades 4–6) were increased in adults (both *p* < 0.05; Figure 1C). In the southern coast, high‐degree sensitization to mites increased, whereas sensitization to Art v decreased in both children and adults (all *p* < 0.05). All degrees of sensitization to *A. alternata* significantly decreased in children (*p* < 0.01; Figure 1D).

Comparison of sensitization rates in patients with different disease phenotypes by stratification to age groups.

**FIGURE 2:**
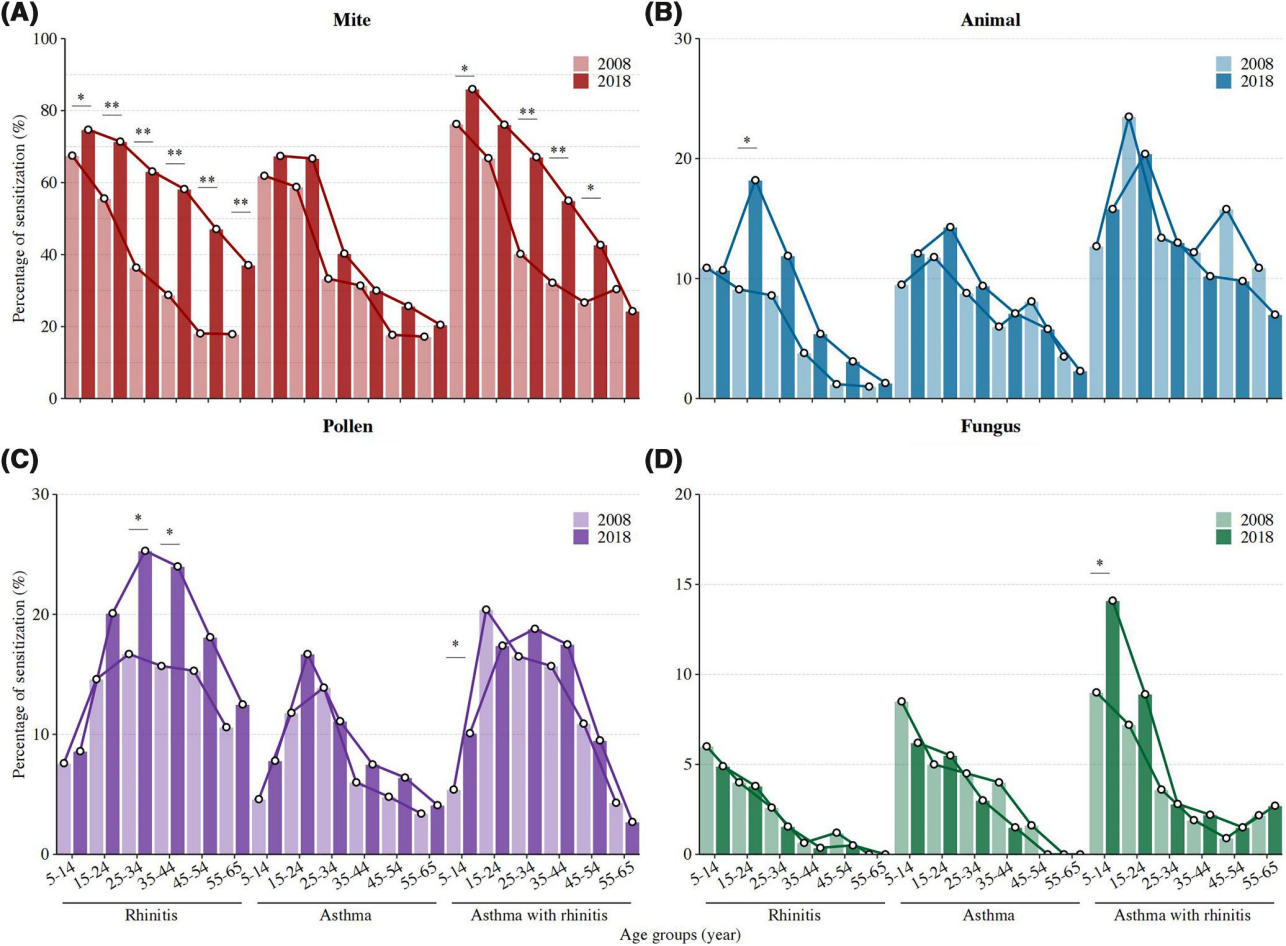
Distribution of sensitizations to different groups of allergens in patients with rhinitis, asthma, and asthma with rhinitis by stratification to age groups between 2008 and 2018. Results for each are based on data from 2008 (2322 participants, rhinitis *n* = 810, asthma *n* = 617, asthmatics with rhinitis *n* = 895) and 2018 (2798 participants, rhinitis *n* = 1871, asthma *n* = 140, asthmatics with rhinitis *n* = 787). *p < 0·05, **p < 0·01

As in 2008, mites were still the most important allergen in 2018, especially for the youngest group. Sensitization to mite increased in nearly all age groups among individuals with rhinitis alone as well as in those with rhinitis and asthma (all *p* < 0.05; Figure 2A). Patients with rhinitis had significantly higher positive rates of pollen sensitization in 2018, peaking between 25 and 44 years (*p* < 0.05; Figure 2C). In 2018, the positive rate of animal sensitization was twofold higher in the 15‐ to 24‐year‐old rhinitis group (*p* < 0.05). In addition, the fungus sensitization rate was increased in children with rhinitis and asthma (*p* < 0.05; Figure 2B and D).

### 5.4 Association of changes in climate or *Artemisia desertorum* planting and sensitization

**FIGURE 3:**
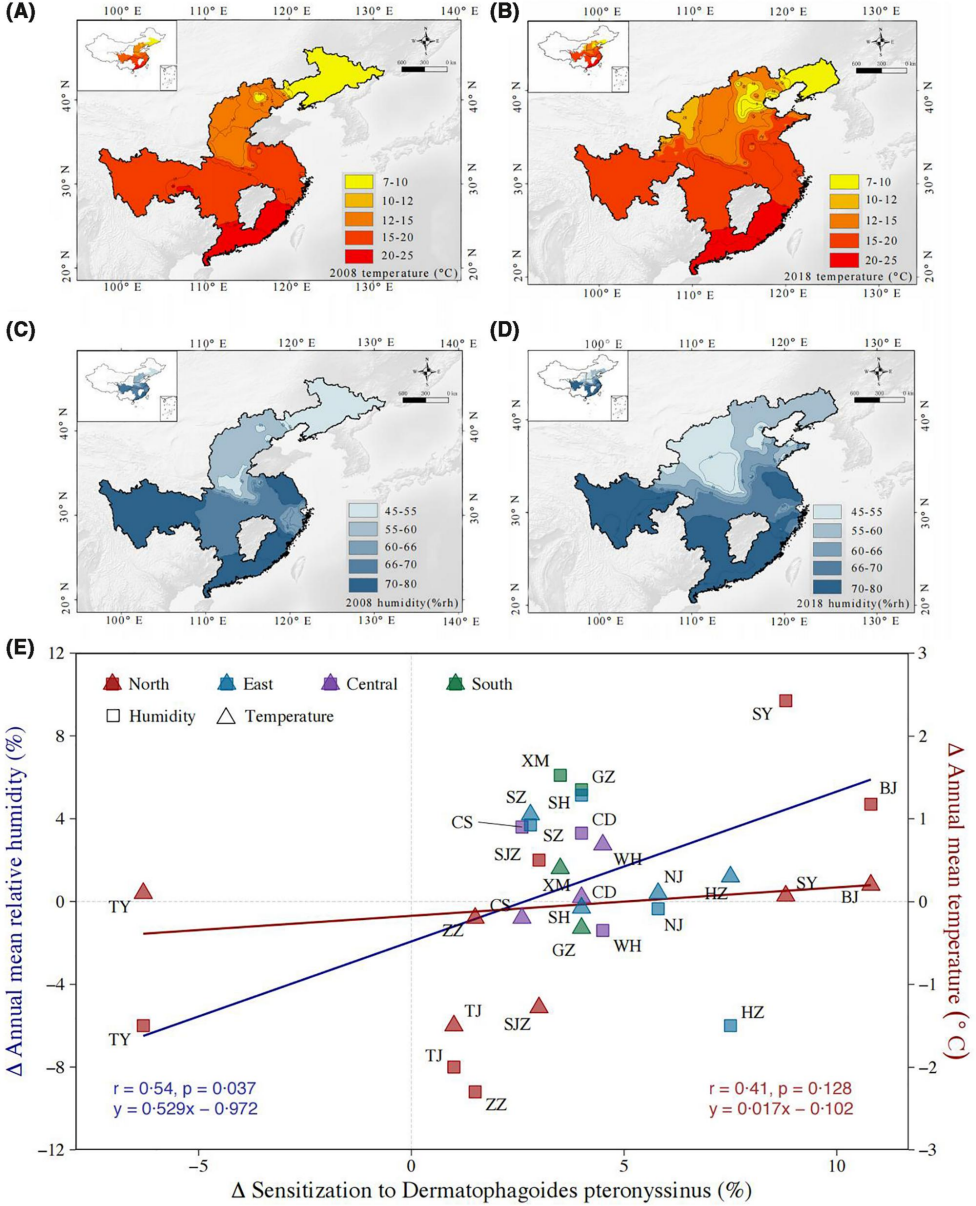
Correlation between climate change and sensitization to *Dermatophagoides pteronyssinus* between 2008 and 2018**.** The depth of color indicates the extent of mean annual temperature (2008 in (A), 2018 in (B)) and mean annual relative humidity (2008 in (C), 2018 in (D)) of the provinces where the study centers enrolled in the study are located. The contour line in the map indicates the numerical value of the extent. The Spearman rank correlation coefficients indicate the associations of changes in temperature and humidity with the sensitization to *D. pteronyssinus* in the 15 cities of the above study centers (E). The spot colors designate: red, northern cities; green, eastern cities; yellow, central cities; blue, southern cities; ∆, Value_2018_−Value_2008_. City abbreviations: Beijing, BJ; Tianjin, TJ; Zhengzhou, ZZ; Shenyang, SY; Shijiazhuang, SJZ; Taiyuan, TY; Shanghai, SH; Nanjing, NJ; Hangzhou, HZ; Suzhou, SZ; Chengdu, CD; Wuhan, WH; Changsha, CS; Guangzhou, GZ; Xiamen, XM

The mean temperature in urban areas ranged from 11.3°C in the north to 22.0°C in the south in 2008 (Figure 3A), while in 2018, it ranged from 11.4°C in the north to 22.3°C in the south (Figure 3B). The mean annual relative humidity ranged from 52.1% in the north to 72.9% in the south in 2008 (Figure 3C), and in 2018, ranged from 55.7% in the north to 77.3% in the south (Figure 3D). The linear correlation model showed that the increase in humidity was associated with the increase in *D. pteronyssinus* sensitization (*r* = 0.54, *p* = 0.037), while no association was observed between change in temperature and *D. pteronyssinus* sensitization (*p* = 0.128; Figure 3E).

**FIGURE 4:**
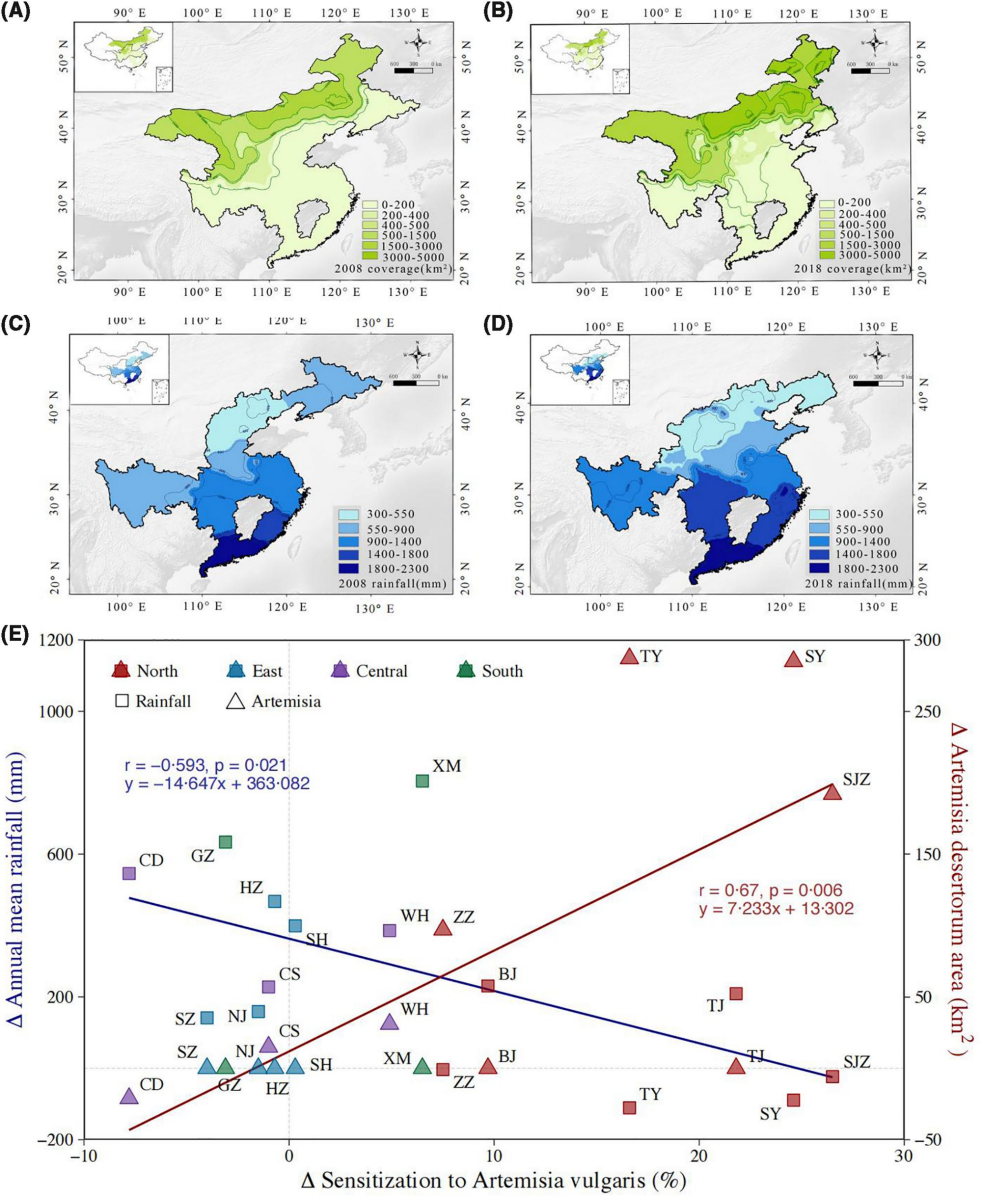
Correlation of climate change and *Artemisia desertorum* planting area with the sensitization to *Artemisia vulgaris* between 2008 and 2018. The depth of color indicates the extent of *A. desertorum* planting area (2008 in (A), 2018 in (B)) and mean annual rainfall (2008 in (C), 2018 in (D)) of the provinces where the study centers enrolled in the study are located. The contour line in the map indicates the numerical value of the extent. The Spearman rank correlation coefficients indicate the association of changes in *A. desertorum* planting area and rainfall with the sensitization to *A. vulgaris* in the 15 cities of the above study centers (E). The spot colors designate: red, northern cities; green, eastern cities; yellow, central cities; blue, southern cities; ∆, Value_2018_−Value_2008_. City abbreviations: Beijing, BJ; Tianjin, TJ; Zhengzhou, ZZ; Shenyang, SY; Shijiazhuang, SJZ; Taiyuan, TY; Shanghai, SH; Nanjing, NJ; Hangzhou, HZ; Suzhou, SZ; Chengdu, CD; Wuhan, WH; Changsha, CS; Guangzhou, GZ; Xiamen, XM

The mean annual rainfall ranged from 495.3 mm in the north to 1887.8 mm in the south in 2008 (Figure 4C), while in 2018, rainfall ranged from 587.8 mm in the north to 2015.2 mm in the south (Figure 4D). The core territory of *A. desertorum* was located in northwest China in 2008 and 2018. However, four northeastern provinces where the study cities were located showed a trend towards increased surface coverage by *A. desertorum* zonation over 10 years (Shenyang in Liaoning from 75 to 330 km^2^, Taiyuan in Shanxi from 60 to 317 km^2^, Shijiazhuang in Hebei from 98 to 285 km^2^, and Zhengzhou in Henan 43–135 km^2^). Several central provinces (Wuhan in Hubei from 5 to 36 km^2^, Changsha in Hunan from 10 to 25 km^2^) also showed a slight trend to an extended planting area, while one location showed a decrease trend in coverage (Chengdu in Sichuan from 41 to 22 km^2^; Figure 4A and B). There was no *A. desertorum* planting in eastern China and the southern coast. A significant positive correlation was observed between the increase in *A. vulgaris* sensitization and expansion of *A. desertorum* planting area (*r* = 0.67, *p* = 0.006). Not surprisingly, a significant negative correlation was observed between the increase in *A. vulgaris* sensitization and the decrease in rainfall (*r* = −0.59, *p* = 0.021; Figure 4E).

## 6 DISCUSSION

The present study covered geographically dispersed regions in China, had a large sample size, and included a range of allergens analyzed using sIgE‐mediated sensitization prevalence over the last decade. The data of this study are the first from China to show that the sensitizations to almost all indoor and outdoor aeroallergens increased over time. House dust mites remained the highest prevalent allergens in individuals with rhinitis and/or asthma. Adults with rhinitis, aged 25–44 years, sensitized to pollens, and individuals with rhinitis, aged 15–24 years, sensitized to animals, increased substantially. Fungus sensitization increased in children with rhinitis and asthma. There was regional heterogeneity in the prevalence rates of allergen sensitization. The prevalence of *D. pteronyssinus* was the highest in southern region (79.2%), while the prevalence of *A. vulgaris* was highest in the north (30.3%). Changes in climate and an increase in *A. desertorum* planting area were associated with the upward trend of sensitization to *D. pteronyssinus* and *A. vulgaris*.

Increased sensitization to common inhaled allergens was seen in other countries including Germany and Korea,^13^, ^14^ which were in agreement with the results of the present study, suggesting a worldwide trend. These changes in sensitization rates may be attributed to the adoption of modern lifestyle, economic development, as well as climate change which increased allergenicity of pollen.^15^ Our data showed an upward trend in *A. vulgaris* sensitization rates reaching 30.3% in northern China, a threefold higher rate compared with that in 2008. Weed pollen, especially that of *A. vulgaris*, is a major outdoor allergen in northern China.^16^ We noted a link between expanded planting of *A. desertorum* and sensitization rates. The increased expanse of this plant is attributed to its use as a wind‐break and as a means of limiting sand displacement in the desert steppe of northwest China. Although the core distribution area is in Inner Mongolia, Gansu, and Shanxi, recent data showed a tendency for spreading of the plant to the northeast (Jilin, Heilongjiang, Liaoning, and Hebei).^17^ The prevalence of allergic respiratory diseases with sensitization to *Artemisia* was increased dramatically in another study.^7^ It is interesting to speculate if *A. desertorum* plays a role in sensitization to *A. vulgaris*. Unfortunately, allergen testing for *A. desertorum* is not available in China and other countries, but those sensitized against *A. vulgaris* might be more likely to respond to *A. desertorum* due to the cross‐reactivity in highly conserved protein of all pollen species.^18^ Additionally, we discovered that adults with rhinitis, aged 25–44 years, were more likely to be influenced by pollens in 2018. The average time an individual spends outdoors will obviously impact their pollen exposure. Interestingly, we observed a negative association between *Artemisia* sensitization and the change in rainfall. Indeed, rainfall may function to clear airborne pollens.^19^ While not assessed specifically in the present study, others noted a vegetation greening trend across China over the past 3 decades.^20^ Further, living in close proximity to areas with high grass coverage adversely effected respiratory health of children.^21^

Recent data noted increasing numbers of patients with mite sensitization in local regions during the last 5 years, while the sensitization rate for mites was much higher in adolescents (91.4%).^22^ As shown herein, allergy to mites increased significantly in individuals with rhinitis with or without asthma, especially in children living in southern China. This group had the highest level of *D. pteronyssinus* sIgE (79.2%). We reported that there was little change in the environmental temperature, while mean humidity increased from 2008 to 2018. Meteorological variations, such as changes in relative humidity, are a critical factor for mite prevalence, with higher concentrations found in damp places.^23^, ^24^ We found an association between the increasing sensitization to *D. pteronyssinus* and increasing humidity in a number of urban centers. Ambient humidity alters room microclimate and mite prevalence in different geographical locations.^25^ Additionally, increased urbanization and human activity is associated with greater atmospheric humidity.^26^ Population growth in urban centers is also associated with increased use of air conditioning, which is itself an important risk factor for allergies.^11^ Moreover, as lifestyle changes, people spend more time indoors, which also drives the increase in sensitization to dust mites.

We also found that pet allergens increased in teenagers with rhinitis. Still, the prevalence of sensitization to cat or dog dander varied among the four zones in China. Geographic variation in the sensitization to furry animals was attributed to early life exposure and rate of pet ownership.^27^, ^28^ The impact of pet ownership on allergen sensitization was not clear. Exposure to furry animals is not limited to pet owners, but can occur in schools and occupational or leisure environments. Animal allergens can be transferred from one area to another passively.^29^ These considerations should be taken into account when analyzing our results. Many individuals react to Fel d one of cats (dander particles <4.7 μm in diameter) that may remain suspended in the air for several days.^30^ High‐efficiency particulate air cleaning systems reduced airborne Fel d 1 levels by 70%–80%.^31^ This may have accounted for, in part, our finding that cat sensitivity did not significantly change during the study interval.

We showed that sensitization to *A. alternata* was higher in children than in adults, with a specific increase in the central region. Our finding is in accordance with previous studies. Sensitization to *A. alternata* may have a genetic component.^32^ It is suggested that routine *A. alternata* sIgE screening is suitable for children with allergies under 15 years of age. *A. alternata* is a hydrophilic fungus that is active in dampness.^33^ Other factors associated with Alternaria sensitization may include male gender, non‐smoker, living in urban areas, or using new building materials.^34^ *A. alternata* can also cause a plant disorder called leaf spot disease.^35^ This might explain our results that found in northern China, an arid area with high plant density, a high rate of *A. alternata* allergy.

Of note, in our study, the majority of centers that had participated in 2008 also participated in 2018, and were evenly distributed in the four regions of China. The two surveys did not cover patients from the entire country, but the four regions covered accounted for nearly two‐thirds of China and represented the real allergen sensitizations of patients with rhinitis/asthma in mainland China. Study subjects were recruited based on CARRAD guidelines with respiratory allergic diseases diagnosed by a physician and standard diagnostic tests. The methodology of the 2018 survey needed to parallel the 2008 survey, especially the study participants. Therefore, age‐stratified sampling was conducted before comparison to adjust for the differences between regions and age groups. Despite some minor bias in sample size of disease phenotypes, which cannot be completely excluded, we made an effort to ensure the consistency of the two surveys.

Our study also has several limitations. First, the study was not a prospective follow‐up study. Thus, it could not evaluate the incidence of allergic respiratory disease. Second, the sensitization rate of the two studies referred to symptomatic patients, rather than the general population, and thus it was difficult to generalize our results to the entire Chinese population. Third, only considering humidity increases over time cannot determine the exact association between the sensitization rate and urbanization, including population growth and density, household income and other factors. Fourth, dust samples from study patient family members, as a surrogate for evaluating home allergen exposure, were not collected. Fifth, specific molecular indicators for *A. desertorum* sensitization still need to be identified. Finally, although we found an association between *A. desertorum* and *Artemisia* sensitization, dynamic collection and analysis of the first‐line data of pollen storm through pollen‐monitoring stations is warranted to confirm this observation.

## 7 CONCLUSIONS

Using nationwide epidemiological study data, we found that the prevalence of sensitization to house dust mites (*D. pteronyssinus*, *D. farinae*, and *B. tropicalis*), pollens (*P. nigra, A. artemisifolia*, and *A. vulgaris*), and animals (dog dander) in individuals with asthma and/or rhinitis significantly increased in China over the last decade.These findings suggest that policies need to be adjusted to mitigate human‐induced climate change and reduce the artificial planting of *A. desertorum*, to limit sensitization to dust mites and *Artemisia*.

## CONFLICT OF INTEREST

The authors have no conflict of interest to declare.

## AUTHOR CONTRIBUTIONS

Jing Li, Nanshan Zhong, Jianhong Wang, Guihua Song, Hua Xie, Xiaoping Lin, Ruonan Chai, Rongfei Zhu, Yong He, Jun Tang, Junge Wang, Jinghua Yang, Lili Zhi, Lin Wu, Yan Jiang, Xiaoqin Zhou, Dongming Huang, Ning Wang, Rui Xu, Yuan Gao, Zhimin Chen, Jinling Liu, Xiaoli Han, Guolin Tan, Jinzhun Wu, Deyu Zhao, Jianjun Chen, Xiwei Zhang, Mengrong Li, Yuemei Sun, Yi Jiang, Weitian Zhang, Qianhui Qiu, Chuanhe Liu, Jie Yin, Guodong Hao, Huabin Li, Yongsheng Xu, Shaohua Chen, Hua Zhang, Shi Chen, Juan Meng, Dan Zeng, Wei Tang, Chuangli Hao had the idea for and designed the study. Jing Li supervised the study and did the writing review. Wanjun Wang did the data curation and wrote the original draft. All authors contributed to acquisition, analysis or interpretation of data. All authors revised the report and approved the final version before submission.

## Supporting information

Figure S1
