# Supplementary figures and images for: Environmental and sensitization variations among asthma and/or rhinitis patients between 2008 and 2018 in China
Source: Clin Transl Allergy. 2022 Feb 2;12(2):e12116. doi: 10.1002/clt2.12116 (PMC8809046; doi:10.1002/clt2.12116)

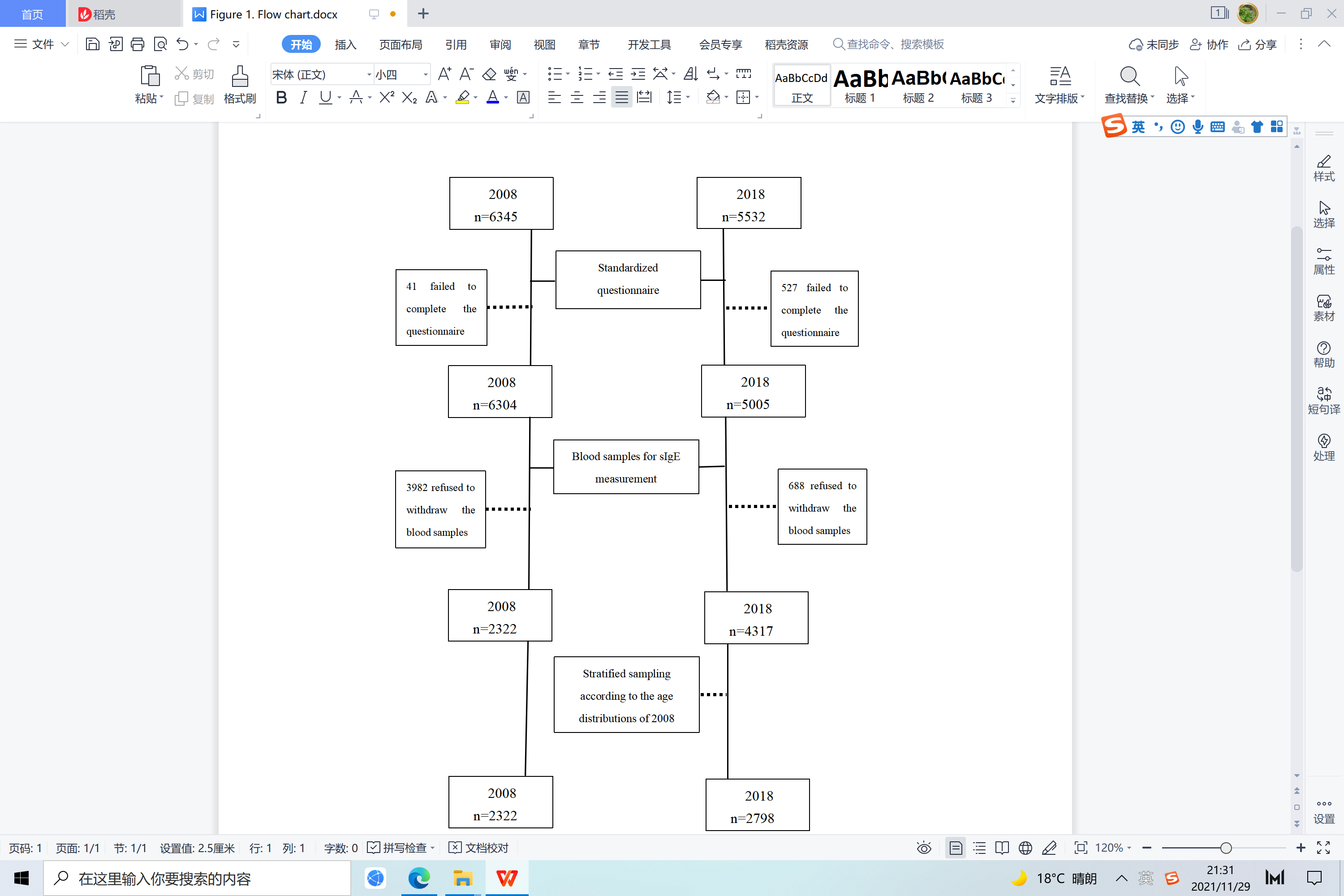


Appendix S1. Flow diagram of data analyzed in this study.

Supplement: Supplementary file 1 — Figure S1 [file CLT2-12-e12116-s001.docx]
